# Role of serum ferritin level on overall survival in patients with myelodysplastic syndromes: Results of a meta-analysis of observational studies

**DOI:** 10.1371/journal.pone.0179016

**Published:** 2017-06-16

**Authors:** Claudia Pileggi, Maddalena Di Sanzo, Valentina Mascaro, Maria Grazia Marafioti, Francesco Saverio Costanzo, Maria Pavia

**Affiliations:** 1 Department of Health Sciences, University of Catanzaro "Magna Græcia", Catanzaro, Italy; 2 Department of Experimental and Clinical Medicine, University of Catanzaro "Magna Græcia", Catanzaro, Italy; Hospital Universitario de Salamanca, SPAIN

## Abstract

**Background:**

The role of serum ferritin (SF) as a prognostic factor has been analyzed in patients with myelodysplastic syndromes (MDS) who have undergone hematopoietic stem cell transplantation (HSCT), but the prognostic role of elevated SF levels is still controversial in lower risk MDS patients. Therefore, we performed a meta-analysis of all available published literature to evaluate whether elevated SF levels are associated with a worse overall survival (OS) among patients with low risk MDS.

**Material and methods:**

A systematic bibliographic search of relevant studies was undertaken in accordance with guidelines for meta-analysis of observational studies in epidemiology. Electronic databases were searched through July 2016 for studies examining the level of SF as a prognostic factor in the adults affected by MDS.

**Results:**

Six articles were included in the meta-analysis. A significant association between OS and SF was achieved for the threshold of SF≥1000 ng/mL, when the only study that used SF cut-off ≥2000 ng/mL was not included in the meta-analysis (RR = 1.33; 95% CI = 1.06–1.67). The estimated risk was 2.58 (95% CI = 1.41–4.74) when a SF cut-off≥500 ng/mL was considered.

**Conclusions:**

Our findings underlined a worse survival in patients with MDS who had higher SF levels. The association was stronger and achieved statistical significance after stratification of analyses in which we excluded cut-offs of SF level considered as outliers. These results suggest that negative impact on OS already exist at SF level ≥500 ng/mL. Prospective studies, are needed to better understand this relationship and, above all, to clarify whether earlier iron chelation therapy could improve patients’ OS.

## Introduction

Serum ferritin (SF) level represents a well-known indicator of the body’s iron stores and inflammation. SF is commonly used as a marker of iron overload (defined as SF ≥1000 ng/mL) [1], but this role has been often questioned, as SF value can be affected by acute infections, inflammations, and even malignant conditions, so that it is also considered as an acute phase protein. Several studies have evaluated tests other than the evaluation of SF level to estimate iron overload, such as Magnetic Resonance Imaging (MRI) and Superconducting Quantum Interference Device (SQUID) biomagnetic liver susceptometry [2–4]; however, SF is still considered the most adequate surrogate parameter for body iron overload concentration, as recently confirmed [4].

Elevated SF levels represent a common condition among patients with myelodysplastic syndromes (MDS) and appears to be largely related to red blood cell (RBC) transfusion-dependence. However, iron overload is also present among many patients after MDS diagnosis and before RBC transfusion [5], being mostly attributed to ineffective erythropoiesis and/or increased gut iron absorption [6].

As for other hematological malignancies [7, 8], several studies have analyzed the role of SF as a prognostic factor in MDS patients reporting that higher SF level was associated with a reduction of both leukemia free survival (LFS) and overall survival (OS) [9,10]. Despite the large number of studies on MDS patients who have undergone hematopoietic stem cell transplantation (HSCT) [1, 11, 12], the prognostic impact of elevated SF levels is still controversial [13], above all in lower risk MDS patients [14].

To gain an insight into the up to date qualitative and quantitative knowledge of the prognostic value of SF levels in MDS patients, who have not undergone HSTC, we performed a meta-analysis of all available published literature. In particular, our aim was to evaluate whether elevated SF levels are associated with a worse OS or with a lower LFS.

## Materials and methods

### Search, study inclusion criteria and quality assessment

A systematic bibliographic search of relevant studies was undertaken from April 2014 until July 2016 in accordance with guidelines for meta-analysis of observational studies in epidemiology (MOOSE). The U.S. National Library of Medicine (MEDLINE) and ISI Web of Science (Science Citation Index) bibliographic databases were searched for studies examining the level of SF as a prognostic factor in the adults affected by MDS. We have used text words as well as medical subject heading (MeSH) terms; for example “serum ferritin”, “myelodysplastic syndrome”, “survival” (S1 Table). These terms and their variants have been used in several combinations. We restricted the search to human studies. All potentially eligible studies were considered for review, limited to English, French, German, Spanish and Portuguese languages. Reference lists of relevant studies and review articles were also searched. If necessary, the corresponding authors were contacted to retrieve further information. The literature retrieval was performed by three independent reviewers (C. P., M. D. S. and V. M.).

Citations selected from the initial search were subsequently screened for eligibility. Studies were included in this meta-analysis if they satisfied the following criteria: (a) primary studies; (b) observational studies (case-controls and cohort studies); (c) enrolment of ≥18 years old patients; (d) provided relative risk (RR), or odds ratio (OR) or hazard ratio (HR) estimates and their 95% confidence intervals (CIs) or sufficient data to calculate these estimates; (f) published until July 2016.

If a study appeared in more than one article, data from the most recent publication were used for statistical analysis.

Studies that involved patients who underwent HSCT and pediatric populations, re-analysis, reviews, letters, meeting abstracts, editorials or commentaries were excluded.

Two of the authors (C. P. and M. D. S.) independently screened titles, abstracts and full papers against the inclusion and exclusion criteria. Where disagreement could not be resolved by discussion, a third author (M. P.) was available. One researcher extracted information from the included studies, with confirmation by a second author.

Two authors (C. P. and V. M.) independently assessed all studies for quality using the validated Newcastle-Ottawa quality assessment scale (NOS) [15] (Table 1).

**Table 1:** Methodological quality assessment (risk of bias) of included studies by Newcastle-Ottawa Scale. For each domain, either a "star" or "no star" is assigned, with a "star" indicating that study design element was considered adequate and less likely to introduce bias. A maximum of two stars can be given for Comparability. A study could receive a maximum of ten stars.

Study	Selection				Comparability	Outcome			Data analysis	Total score
	Exposed cohort	Non-exposed cohort	Ascertainment of exposure	Outcome of interest		Assessment of outcome	Length of follow-up	Adequacy offollow-up		
Chee et al., 2008	*	*	*	-	*	*	*	-	-	6
Cermak et al., 2009	-	*	*	-	**	*	*	*	-	7
Park et al., 2011	*	*	*	*	**	*	*	*	*	10
Kikuchi et al., 2012	*	*	*	-	**	*	*	*	*	9
Komrokji et al., 2012	*	*	*	-	*	*	-	*	*	7
Li et al., 2013	*	*	*	-	*	*	*	*	*	8

The scale must be adapted a priori for use according to the research question and the study topic. The NOS uses a star system in which studies are judged on key domains pertaining to the selection and comparability of study groups, the ascertainment of exposure and outcome, and the duration of follow-up. We modified this scale by integrating a question about statistical evaluation of included studies. For each domain, 1 point (star) was assigned. A study could receive a maximum of 10 points. Studies were classified as high quality if their score was higher than the median.

### Data extraction, outcomes and data analysis

All data from the studies were independently extracted by two of the authors using a designed form. The accuracy of the extracted data was checked by a third reviewer. For each study, the following information was abstracted: (a) first author’s name, year of publication, and country of the population; (b) study design; (c) number of subjects, sex, age range; (d) selected SF levels as a threshold of iron overload; (e) International Prognostic Scoring System (IPSS); (f) eventual RBC transfusion dependency; (g) iron chelator therapy; (h) duration of follow-up; (i) LFS; (j) OS.

All data were extracted from the published papers. If necessary, the corresponding authors were contacted to retrieve further information.

We planned to perform several meta-analyses to evaluate the effect of SF on OS and LFS. Within each study, the risk estimates (RR, OR, HR) were extracted, if available, or calculated and expressed as RRs and 95% CIs. Separate meta-analyses were conducted for 2 possible thresholds of SF: ≥300ng/mL and ≥1000ng/mL. All meta-analyses were carried out using the Der Simonian and Laird random effect model [16] to incorporate heterogeneity within and between studies. If the studies were homogeneous, the Mantel-Haenszel fixed effects model [17] was also used to assess the effect of model assumptions on our conclusions. Statistical heterogeneity was assessed using Cochran Q and I^2^ measure; an I^2^ value above 50% and 75% were predefined as moderate and high heterogeneity [18]. Results of the meta-analyses were expressed as RR and the related 95% CI.

Since LFS was the outcome in only two included studies that could not be pooled, meta-analyses with LFS as outcome were not performed.

The studies included in the meta-analysis differed considerably in several factors such as characteristics of patients, baseline disease severity, transfusion dependence, iron chelating therapy, and follow up period. So in this meta-analysis there was extensive clinical heterogeneity, and therefore subgroup analyses were used to explore heterogeneity.

We performed separate sensitivity analyses by grouping studies that were similar according to the following characteristics: SF cut-off (≥1000 ng/mL or ≥2000 ng/mL for studies that considered SF level ≥1000 ng/mL; ≥500 ng/mL or ≥300ng/mL for studies that considered SF level ≥300 ng/mL); transfusion status (transfused or non-transfused patients); criteria used for MDS diagnosis (WHO or FAB classification). Finally, we performed a meta-analysis to determine the potential impact of the quality of the studies on the results, by pooling only studies with NOS score higher or lower than the median. Univariate meta-regressions were also performed to examine the effect of the previously mentioned factors on the relationship between SF and OS.

As the number of included studies was less than 10, we did not perform statistical tests for funnel plot asymmetry because, in these conditions, the power of the tests to explore publication bias is considered too low to distinguish chance from real asymmetry [19].

All statistical analyses were performed using Stata software program, version 14.1 (Stata Corporation. College Station, TX).

## Results

### Study characteristics

**Fig 1:**
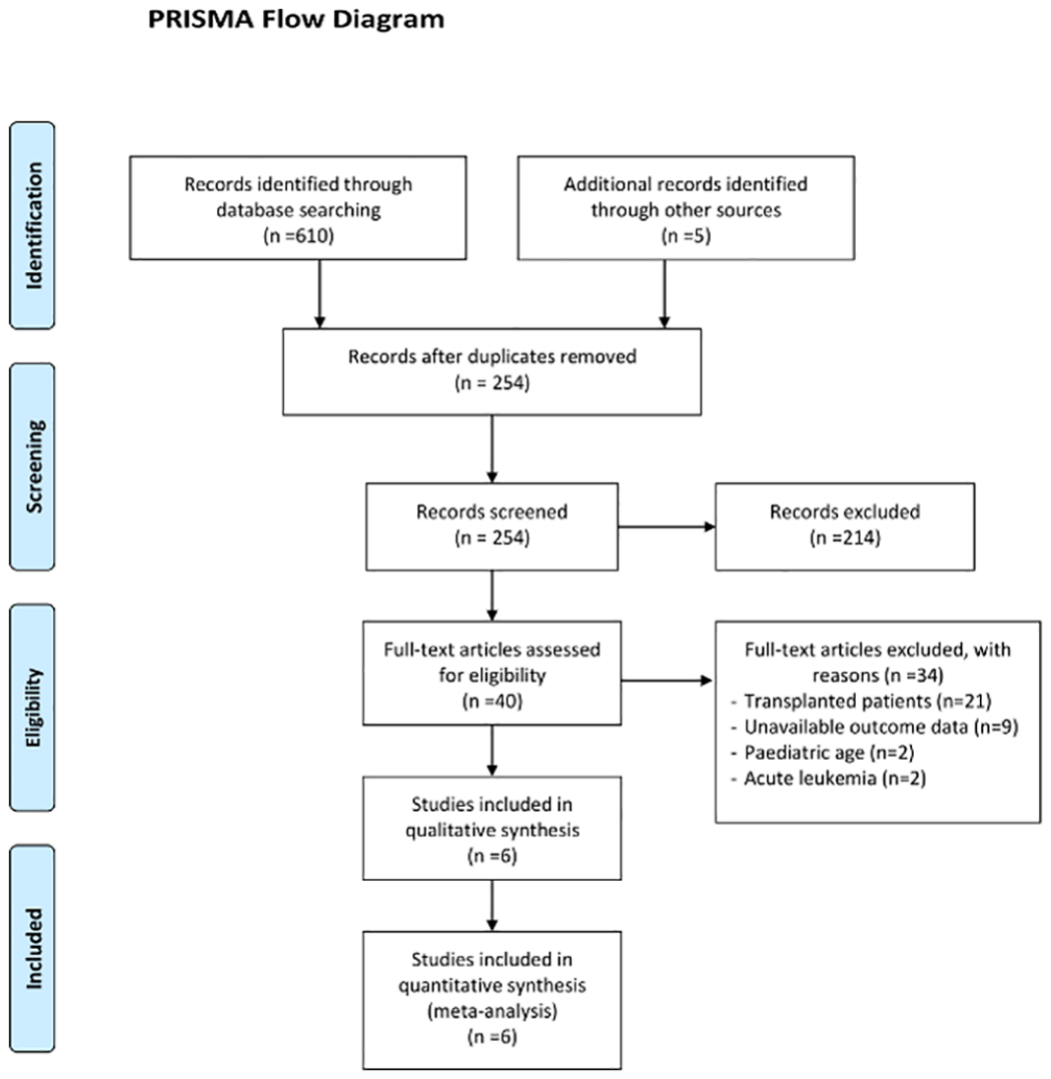
Flow chart of the included studies in the meta-analysis.

Literature searches yielded 615 records. After exclusion of duplicate records and studies that did not fulfil our inclusion criteria, 42 articles were retrieved and reviewed in full text that resulted in the inclusion of 15 studies. Of these, nine studies had no available data on survival: five studies were excluded owing to lack of sufficient data for estimation of RRs and in four no original data could be extracted even after the corresponding authors were contacted to retrieve further information. Finally, six articles met the inclusion criteria and were available for the meta-analysis. The inclusion and exclusion process is summarized in Fig 1.

The characteristics of the eligible studies are outlined in Table 2. All 6 articles were retrospective cohort designs. Three studies were conducted in departments of hematology [20–22], one in a department of internal medicine [23], and two studies gathered information from databases of cancer centers [14, 24]. The principal objective of one study [24] was to examine the role of serum albumin as a prognostic marker for OS in MDS.

**Table 2:** Characteristics of observational studies included in the meta-analysis.

Authors	Country	Sample^a^	Median age, years	Classification used for diagnosis	MDS subtypes	Patients' IPSS	Serum ferritin cut-off (ng/ml)	Exposed/Not exposed	Variables of adjustment	Outcome(RR, 95%CI)	Median follow-up time, months
Chee et al. 2008	USA	77	73	FAB	RARS	Low, IM-1, IM-2	≥1000	14/63	RBC transfusion	OS (1.21, 0.39–3.75)	36
Cermak et al. 2009	Czech Republic	137	49.4	WHO	RA, RARS, RCMD, RS-RCMD, 5q	Low, IM-1, IM-2	≥2000	55/52	RBC transfusion, corticosteroid and/or cyclosporine A therapy, oral iron chelator therapy, multilineage dysplasia	OS (3.57,1.51–8.47)	-
Park et al. 2011	France	318	77	WHO	RA, RARS, RCMD, RS-RCMD, RAEB, 5q	Low, IM-1	≥300	153/165	Hb level, gender, ringed sideroblasts, platelets, MCV, sEPO level, WHO classification	LFS (1.76, 0.86–3.62) OS (0.91, 0.57–1.43)	31
							≥1000	32/286		LFS (1.81, 0.55–5.91) OS (0.85, 0.4–1.81)	
Kikuchi et al. 2012	Japan	47	-	WHO	RC, RCMD, RAEB	Low, IM-1, IM-2, High	≥300	19/28	None	LFS (4.75,0.85–26.51) OS (3.44, 1.14–10.36)	50
							≥500	10/37		LFS (21.16, 2.06–217.1) OS (1.9, 1.03–3.5)	
Komrokji et al. 2012	USA	767	69	WHO	RA, RARS, RCMD, RAEB, MDS-u	Low, IM-1, IM-2, High	≥1000	-	RBC transfusion, age, IPSS	OS (1.4, 1.1–1.9)	35
Li et al. 2013	China	191^2^	50	WHO	RA, RARS, RCMD, RAEB, 5q, MDS-u	IM-1	≥500	74/117^b^	RBC transfusion, age, gender, Hb level, platelets, absolute neutrophil level percentage of bone marrow blasts, karyotipe, cellularity of bone marrow biopsy, grade of bone marrow fibrosis	Total sample OS (3.53, 1.9–6.6) Transfused patients OS (2.88, 1.61–5.13) Non-transfused patients OS (3.36, 1.51–7.49)	21

FAB: French-American-British; WHO: World Health Organization; MDS: Myelodysplastic syndrome; RA: Refractory anemia; RARS: refractory anemia with ring sideroblasts; RC: refractory cytopenia with unilineage dysplasia; RCMD: refractory cytopenia with multilineage dysplasia; RS-RCMD: refractory cytopenia with multilineage dysplasia with ring sideroblasts; RAEB: refractory anemia with excess blasts; 5q: 5q syndrome; MDS-u: MDS unclassified; IPSS: International Prognostic Scoring System. Low (Low risk):0; IM-1(Intermediate risk-1): 0.5–1; IM-2 (Intermediate risk-2): 1.5–2; High (High risk): ≥2.5; RBC: red blood cells; Hb: hemoglobin; MCV: mean corpuscular volume; sEPO: serum erythropoietin; OS: Overall survival; LFS: leukemia free survival.

^a^All studies were designed as retrospective cohort.

^b^The reported number refers to the total sample including both transfused and non-transfused patients. The separate number of subjects included in the transfused and in the non-transfused group, according to the exposure, was not available in the study.

Overall, in the included studies, 1855 participants were recruited. The number of subjects per study varied between 47 and 767 (median 191). Patients' median age ranged from 49.4 to 77 years. In 3 studies, the participants did not receive the iron chelation therapy [14, 22, 23], and one other involved patients who required chelation therapy [21], whilst the remaining studies did not specify patient status related to chelation therapy [20, 24]. The MDS diagnosis was defined according to the WHO classification [25] in all studies except one [20], which was defined by the FAB classification [26]. Risk stratification based on IPSS was performed in all included studies and ranged from low to high risk in two studies [23, 24], while patients with low risk disease were included in the remaining studies.

### Data quality

The mean quality scores of the individual studies using the NOS scale ranged from 6/10 to 10/10 (median 7.5). Scores of each study are reported in Table 1.

The agreement between the two researchers in the first comparison was 89%, with a kappa score of 0.88, and after discussion and detailed review of the articles it was complete (k = 1).

### Meta-analysis

**Fig 2:**
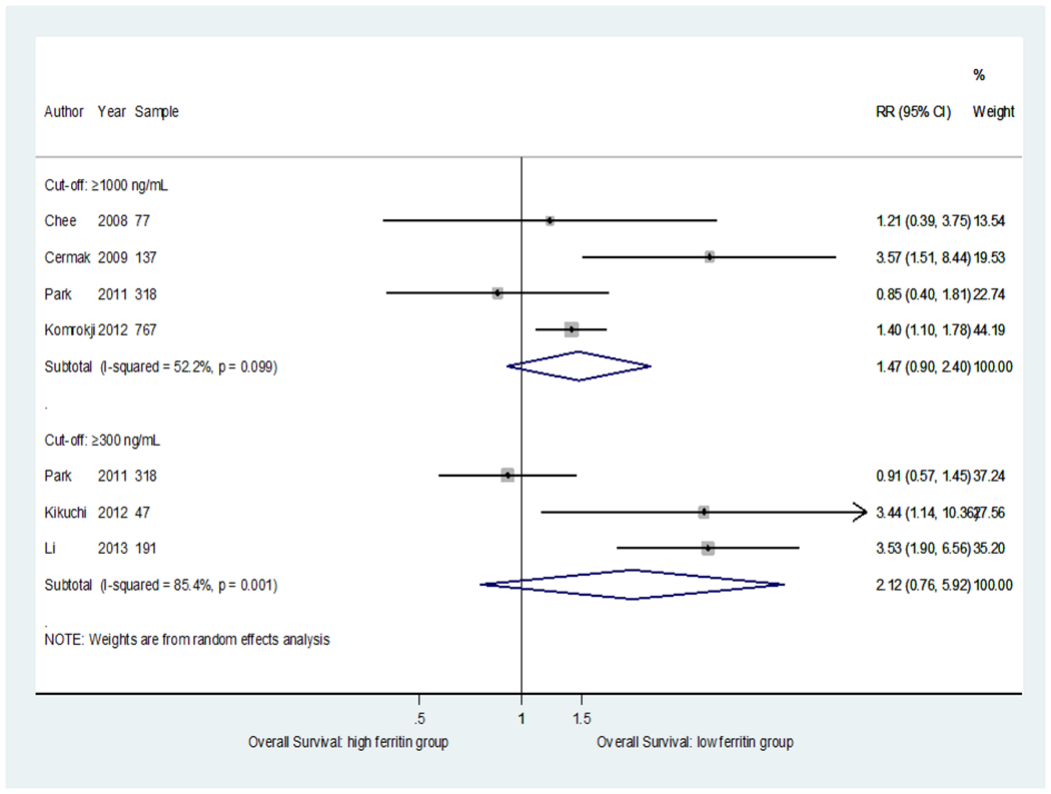
Forest plot of the association of OS and SF according to SF≥1000 ng/ml and SF≥300 ng/ml cut-offs.

The results of the meta-analysis that compared OS among the patients with MDS are shown in Fig 2.

For a threshold of SF≥1000 ng/mL, data from a total of 1299 participants were analyzed; the pooled RR when comparing the highest to the lowest category of SF level and OS was 1.47 (95% CI = 0.9–2.4); heterogeneity: p = 0.099; I^2^ = 52.2%. The overall RR estimates for a threshold of SF≥300 ng/mL, from a total of 556 participants, was 2.12 (95% CI = 0.76–5.92); heterogeneity: p = 0.001; I^2^ = 85.4%.

**Fig 3:**
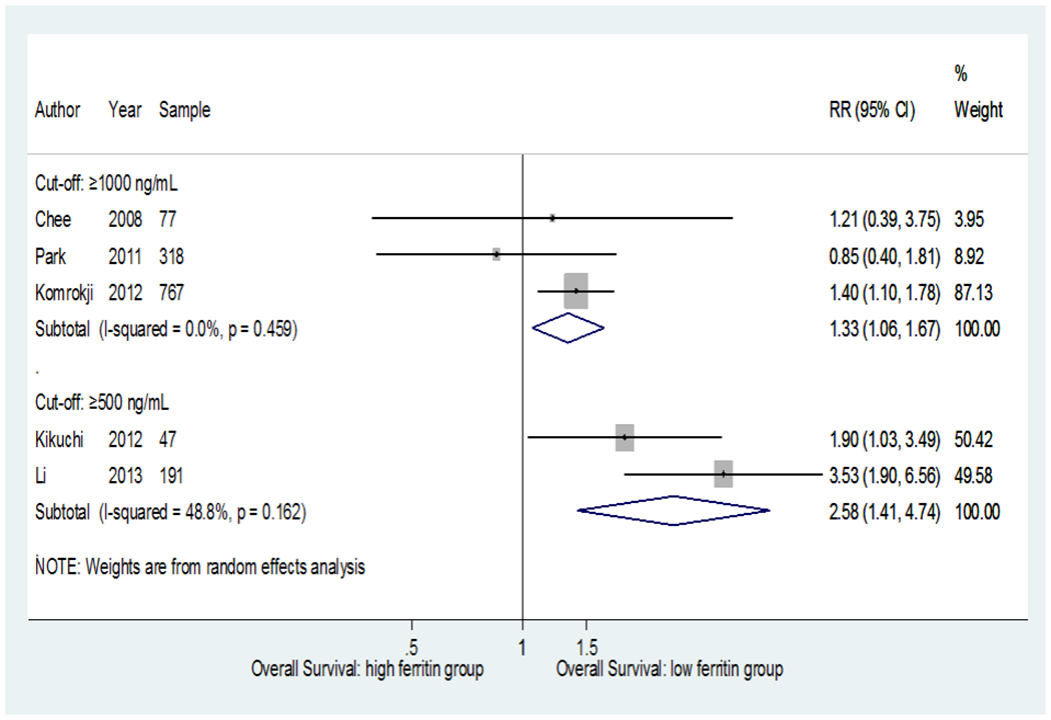
Forest plot of the subgroup analyses of the association of OS and SF according to SF≥1000 ng/mL and SF≥500 ng/mL cut-offs.

To examine the stability of the primary results, we carried out sensitivity analyses that substantially confirmed the primary meta-analysis. In particular, for the threshold of SF≥1000 ng/mL, a significant association between OS and SF was achieved when the only study that used an SF cut-off ≥2000 ng/mL was not included in the meta-analysis (RR = 1.33; 95% CI = 1.06–1.67), and heterogeneity disappeared (p = 0.46; I^2^ = 0%) (Fig 3).

A significant association and a reduction of the heterogeneity were also reached when the analysis was restricted to studies that considered a SF cut-off ≥500 ng/mL. In this case the estimated risk was 2.58 (95% CI = 1.41–4.74; p = 0.162; I^2^ = 48.8%) (Fig 3).

The other sensitivity analyses (Table 3), that took into account the MDS classification (the use of the WHO or the FAB classification) and the quality of studies (low and high quality studies), showed a little reduction in the heterogeneity; the only exception was the analysis that pooled data of non-transfused patients only, in the group with an SF level ≥1000 ng/mL that showed no heterogeneity.

**Table 3:** Summary of overall and subgroup analysis describing the association between OS and SF levels in patients with MDS.

	N. studies	N. patients	Overall RR	95% CI	Heterogeneity test (p; I^2^%)
**Serum ferritin level ≥1000 ng/mL**					
All studies	4	1299	1.47	0.9–2.4	0.099;52.2
Transfused patients	2	904	2.03	0.83–4.99	0.04;76.3
Not-transfused patients	2	395	0.95	0.51–1.77	0.611;0
WHO classification	3	1222	1.54	0.83–2.85	0.045;67.8
FAB classification	1	77	1.21	0.39–3.75	-
Low quality studies	3	981	1.75	0.96–3.18	0.114;54
High quality studies	1	318	0.85	0.4–1.81	-
**Serum ferritin level ≥300 ng/mL**					
All studies	3^a^	556	2.12	0.76–5.92	0.001;85.4
Transfused patients	1	Not reported	2.88	1.61–5.13	-
Not-transfused patients	3	365^b^	1.7	0.81–3.6	0.012;77.3
Low quality studies	1	191	3.53	1.9–6.56	-
High quality studies	2	365	1.28	0.62–2.63	0.06;71.7

^a^The total studies included in overall meta-analysis may be lower than the sum of stratified meta-analyses because the total number of patients had to be split to be included in more than one stratified meta-analysis. In any case, no patient was included more than once in any of the meta-analyses.

^b^Reported number includes only patients from two studies due to the missing data of the third study included in the meta-analysis [22].

Finally, the results of the meta-regression analysis showed that none of the study characteristics, such as patients’ age, RBC transfusions, iron chelation therapy, quality of studies, substantially modified the pooled estimates (S2 Table).

All presented data were derived from random effects models.

## Discussion

The present study is the first meta-analysis summarizing the positive association between SF levels and OS in patients with MDS that have not undergone HSCT.

Our findings underlined a worse survival in MDS patients with higher SF level. The association was stronger and achieved statistically significance when SF 1000 ng/mL and SF 500 ng/mL pre-specified cut-offs were considered.

With respect to the SF cut-off of 1000 ng/mL, our results showed that the estimated survival was 33% higher in patients with an SF level <1000 ng/mL compared with those with SF level >1000 ng/mL. This was an expected result, considering that most of the guidelines on management of MDS suggest to consider iron chelation therapy when the SF level exceeds 1000 ng/mL [26–30]. This value is considered to be better, compared with higher levels, in the identification of the population at risk of iron-related organ complications, such as cardiac or liver dysfunction. It is reasonable to assume that much higher than 1000 ng/mL SF levels may reflect a more severe underlying disease and not an adverse effect of iron overload, as previously hypothesized by Armand et al. [13].

Considering the debate about the appropriate SF level to start chelation therapy, as a novel finding, our meta-analysis suggests that a lower OS may be related to an even lower than 1000 ng/mL SF level, that is 500 ng/mL. Indeed, results of the pooled analysis of studies that considered an SF cut-off of 500 ng/mL, showed that the estimated survival was 2.58 times higher in patients with an SF level <500 ng/mL than in those with an SF level ≥500 ng/mL. This finding most likely suggest that the prognostic role of SF levels on OS of MDS patients can be set at a lower than the established 1000 ng/mL level. Therefore, our meta-analysis has provided data specifically concerning the prognostic importance of a higher than 500 ng/mL SF level, that has not been previously described.

Although the main cause of iron overload in MDS is RBC transfusion, most of the patients included in the pooled analysis [22, 23] had not received regular blood transfusion. Observed iron excess was probably a result of ineffective erythropoiesis and of a consequent tissue hypoxia which increases gastrointestinal iron absorption and release of stored iron [5, 31]. Although many aspects of the fundamental biology of SF remain unclear, a growing number of roles have been attributed to extracellular ferritin, including a severe suppression of the proliferative capacity of the bone marrow [31]. This effect appears early and it is also present in moderate iron overload, leading to a worsening of the preexisting ineffective erythropoiesis [31]. Therefore, an important implication of our, could be that in MDS patients with an SF level of ≥500 ng/mL treatment may be used to improve this ineffective erythropoiesis leading to a possible survival advantage.

### Strengths and limitations

To the best of our knowledge, this is the first meta-analysis that showed, in MDS patient, that negative impact on OS already exists at an SF level of ≥500 ng/mL.

This study has several strengths. We performed a careful meta-analysis using three different data sources. The authors reviewed the data independently, thereby minimizing the risk of bias in selecting studies or missing data. We also performed meta-regression analyses to identify sources of heterogeneity that commonly occur during meta-analyses of observational data.

Possible limitations take account of heterogeneity among studies performed in patients with different characteristics related to factors that might have adversely influenced OS (e.g. baseline disease severity, transfusion dependence, iron chelation therapy). We thought that the different SF cut-offs and the transfusion dependence could explain the statistical heterogeneity that has been found between the studies’ results and, indeed, heterogeneity disappeared when we performed subgroup analyses of studies that considered SF cut-offs of ≥1000 and ≥500 ng/mL and in non-transfused patients (for SF ≥1000 ng/mL). However, we were not able to eliminate heterogeneity in the other sensitivity analyses. Therefore, results must be interpreted with caution, taking also into account the difficulty found in the extraction of data that highlights a need for standardization in the conduction of studies and, above all, in the reporting of results.

In conclusion, evidence regarding the prognostic role of moderate iron overload suggests a negative effect toward OS among MDS patients. Prospective studies, are needed to better understand this relationship and, above all, to clarify whether earlier iron chelation therapy could improve patients’ OS.

## Supporting information

S1 Table Electronic search strategy on PubMed. (DOCX)

S2 Table Results from univariate meta-regression analyses relating the effect of several variables on the relationship between SF and OS. (DOCX)

S1 File PRISMA checklist. (DOC)
