# Supplementary material for: Role of serum ferritin level on overall survival in patients with myelodysplastic syndromes: Results of a meta-analysis of observational studies
Source: PLoS One. 2017 Jun 16;12(6):e0179016. doi: 10.1371/journal.pone.0179016 (PMC5473533; doi:10.1371/journal.pone.0179016)
Supplement: S1 Table — (DOCX) [file pone.0179016.s001.docx]

| Search | Search terms | Hits |
| --- | --- | --- |
|  |  |  |
| 1 | ("ferritins"[MeSH Terms] OR "ferritins"[All Fields] OR "ferritin"[All Fields]) AND (("myelodysplastic syndromes"[MeSH Terms] OR ("myelodysplastic"[All Fields] AND "syndromes"[All Fields]) OR "myelodysplastic syndromes"[All Fields] OR ("myelodysplastic"[All Fields] AND "syndrome"[All Fields]) OR "myelodysplastic syndrome"[All Fields]) OR mds[All Fields]) | 351 |
|  |  |  |
| 2 | serum "serum"[MeSH Terms] OR "serum"[All Fields]  ferritin "ferritins"[MeSH Terms] OR "ferritins"[All Fields] OR "ferritin"[All Fields]  myelodysplastic syndrome "myelodysplastic syndromes"[MeSH Terms] OR ("myelodysplastic"[All Fields] AND "syndromes"[All Fields]) OR "myelodysplastic syndromes"[All Fields] OR ("myelodysplastic"[All Fields] AND "syndrome"[All Fields]) OR "myelodysplastic syndrome"[All Fields] | 200 |
|  |  |  |
| 3 | serum "serum"[MeSH Terms] OR "serum"[All Fields]  ferritin "ferritins"[MeSH Terms] OR "ferritins"[All Fields] OR "ferritin"[All Fields]  myelodysplastic syndrome "myelodysplastic syndromes"[MeSH Terms] OR ("myelodysplastic"[All Fields] AND "syndromes"[All Fields]) OR "myelodysplastic syndromes"[All Fields] OR ("myelodysplastic"[All Fields] AND "syndrome"[All Fields]) OR "myelodysplastic syndrome"[All Fields] OR "mds"[All Fields]  survival "mortality"[Subheading] OR "mortality"[All Fields] OR "survival"[All Fields] OR "survival"[MeSH Terms] | 55 |

**Table S1.** Electronic search strategy on PubMed
