# Supplementary material for: Role of serum ferritin level on overall survival in patients with myelodysplastic syndromes: Results of a meta-analysis of observational studies
Source: PLoS One. 2017 Jun 16;12(6):e0179016. doi: 10.1371/journal.pone.0179016 (PMC5473533; doi:10.1371/journal.pone.0179016)
Supplement: S2 Table — (DOCX) [file pone.0179016.s002.docx]

| **Variable** | **Cut-off: ≥1000 ng/mL** | | | | **Cut-off: ≥300 ng/mL** | | | |
| --- | --- | --- | --- | --- | --- | --- | --- | --- |
|  | **Regression Coefficient** | **SE** | ***p*** | **Heterogeneity**  **test (*I^2^*, %)** | **Regression Coefficient** | **SE** | ***p*** | **Heterogeneity**  **test (*I^2^*, %)** |
| Patients’ age  (Continuous) | -0.05 | 0.02 | 0.13 | 0 | -0.05 | 0.01 | 0.054 | 0 |
| RBC transfusion  (Not-transfused patients=0; Transfused patients=1) | 0.68 | 0.57 | 0.354 | 55.30 | 0.64 | 0.49 | 0.276 | 66.74 |
| Iron chelation therapy  (No = 0; Yes = 1) | *insufficient observations* | | | | | | | |
| Quality of studies  (Low = 0; High = 1) | -0.72 | 0.63 | 0.373 | 53.99 | -0.96 | 0.32 | 0.06 | 20.41 |

**Table S1.** Results from univariate meta-regression analyses relating the effect of several variables on the relationship between SF and OS.

SF: Serum Ferritin; OS: Overall survival; RBC: Red Blood Cell.
